# Exploring the Impacts of Genotype-Management-Environment Interactions on Wheat Productivity, Water Use Efficiency, and Nitrogen Use Efficiency under Rainfed Conditions

**DOI:** 10.3390/plants10112310

**Published:** 2021-10-27

**Authors:** Raheel Osman, Muhammad Naveed Tahir, Syed Tahir Ata-Ul-Karim, Wajid Ishaque, Ming Xu

**Affiliations:** 1 Henan Key Laboratory of Earth System Observation and Modeling, Henan University, Kaifeng 475004, China; raheel_osman@yahoo.com; 2 College of Geography and Environmental Science, Henan University, Kaifeng 475004, China; 3 Department of Agronomy, PMAS-Arid Agriculture University, Rawalpindi 46300, Pakistan; naveed@uaar.edu.pk; 4 Graduate School of Agricultural and Life Sciences, The University of Tokyo, 1-1-1 Yayoi, Bunkyo, Tokyo 113-8657, Japan; ataulkarim@g.ecc.u-tokyo.ac.jp; 5 Nuclear Institute for Agriculture and Biology, Faisalabad 38000, Pakistan; raoumar05@yahoo.com

**Keywords:** N fertilization, semi-arid region, heat stress, agronomic traits, favorable growing season, unfavorable growing season

## Abstract

Wheat production under rainfed conditions is restrained by water scarcity, elevated temperatures, and lower nutrient uptake due to possible drought. The complex genotype, management, and environment (G × M × E) interactions can obstruct the selection of suitable high yielding wheat cultivars and nitrogen (N) management practices prerequisite to ensure food security and environmental sustainability in arid regions. The agronomic traits, water use efficiency (WUE), and N use efficiencies were evaluated under favorable and unfavorable weather conditions to explore the impacts of G × M × E on wheat growth and productivity. The multi-N rate (0, 70, 140, 210, and 280 kg N ha^−1^) field experiment was conducted under two weather conditions (favorable and unfavorable) using three wheat cultivars (AUR-809, CHK-50, and FSD-2008) in the Pothowar region of Pakistan. The experiments were laid out in randomized complete block design (RCBD), with split plot arrangements having cultivars in the main plot and N levels in the subplot. The results revealed a significant decrease in aboveground biomass, grain yield, crop N-uptake, WUE, and N use efficiency (NUE) by 15%, 22%, 21%, 18%, and 8%, respectively in the unfavorable growing season (2014–2015) as compared to favorable growing season (2013–2014) as a consequence of less rainfall and heat stress during the vegetative and reproductive growth phases, respectively. FSD-2008 showed a significantly higher aboveground biomass, grain yield, crop N-uptake, WUE, and NUE as compared to other wheat cultivars in both years. Besides, N_140_ appeared as the most suitable dose for wheat cultivars during the favorable growing season. However, any further increase in N application rates beyond N_140_ showed a non-significant effect on yield and yield components. Conversely, the wheat yield increased significantly up to 74% from N_0_ to N_70_ during the unfavorable growing season, and there was no substantial difference between N_70_–N_280_. The findings provide opportunities for maximizing yield while avoiding excessive N loss by selecting suitable cultivars and N application rates for rainfed areas of Pothowar Plateau by using meteorological forecasting, amount of summer rainfall, and initial soil moisture content.

## 1. Introduction

Pakistan ranked eighth in global wheat production (24.3 million tons) during 2019. However, wheat production in Pakistan is gradually decreasing due to global climate change, and wheat production in the country decreased by 5.5% in 2019 compared to the national average during the past five years [1]. Approximately 80% of the country’s land area is situated in arid and semi-arid climate zones, which comprise nearly 25% of the total cultivated area (23.3 million hectares) of Pakistan [2]. Besides, Pakistan is ranked fifth among the most vulnerable countries to climate change worldwide [3]. The average yield gap of wheat in rainfed regions of Pakistan is approximately 66% [2]. Crop production in the rainfed areas of Pakistan is often jeopardized by rising temperatures during the wheat growth period, which is further expected to rise by 2–3 °C by 2050 [4]. Water scarcity, infertile soils, and lack of appropriate cultivars are other main constraints for rainfed agriculture [2,5,6]. Besides, the unpredictability of precipitation makes the nitrogen (N) scheduling both in terms of rate and time more challenging in these regions. N fertilizer application rate and timing are critical for optimizing yield. However, owing to the high risk posed by climatic uncertainties and the high cost of N fertilizer, the farmers in rainfed regions practice low input agriculture (insufficient N fertilizer application) as risk management [2,6,7]. N supply and crop N demand should be synchronized with an adequate water supply as low N availability reduces water use efficiency (WUE) and widens the yield gap between actual and water-limited yield potential [8]. Consequently, the synchronization between crop water and N supply and demand is imperative from an agronomic and economic perspective to ensure sustained crop production under rainfed environments [6]. Despite the positive association between N application, moisture availability, and yield improvements [9,10], the interaction between N management and moisture availability has not received much attention [11]. Besides, the N scheduling in rainfed areas, it is recommended to consider the water availability and crop demand [7]. Nevertheless, N application rates are generally chosen based on logistics rather than understanding of crop demand, soil moisture conditions, and rainfall predictions in rainfed areas [11].

Crop transpiration and soil evaporation both contribute to crop’s seasonal water use, accounting for 88% and 12% of crop evapotranspiration, respectively [12]. The water use efficiency (WUE; the ratio of grain yield to evapotranspiration) and nitrogen use efficiency (NUE; the ratio of grain yield to available N from the soil) are considered as critical parameters in defining crop productivity under water-limited environments [13]. In fertilized fields, NUE is defined as the ratio of additional grain yield to fertilizer N input. This calculation disregards mineral nitrogen stored in the soil or derived from soil organic matter mineralization, which may account for some of the variations in NUE [13]. However, the simultaneous quantification of WUE and NUE using various cultivars and management practices has been rarely quantified, particularly under water-limited environments. Moreover, Sadras, et al. [6] emphasized the need to investigate the crops, soils, and growing conditions in combination where additional N can improve soil-water uptake.

Heat stress events during the reproductive phase of wheat due to global climate change are gradually increasing [14]. According to Sadras, et al. [6], heat stress, water scarcity, and N deficiency combine to compromise wheat growth and development in arid areas. Therefore, identifying the suitable cultivar and optimal N fertilization management technique would necessitate field experiments conducted in favorable and unfavorable weather conditions. Costa, et al. [9] found that single and split N applications are most effective for enhancing wheat production under favorable and unfavorable cultivation years, respectively. Years have been classified as favorable or unfavorable for wheat farming based on the amount and distribution of rainfall and air temperature [15]. Cossani and Sadras [16] reported that wheat yield is affected by elevated temperatures, N, cultivar, environment, and interactions. For instance, high temperatures in unfertilized plants reduce yield and aboveground biomass by 17% and 15%, respectively, compared to fertilized crops [16]. But, in this study, the maximum temperature did not exceed the threshold level (>32 °C), and open-top chambers were used to increase the temperature. Additionally, comparing the impact of N application rates and genotypes on wheat yield under favorable and unfavorable weather conditions has been rarely investigated [9]. Field experiments that did not encounter adverse weather conditions are unlikely to reflect crop response information accurately for unfavorable weather conditions. Further, data from actual field conditions is scarce, where low and erratic rainfall, high temperatures, low soil nutrient availability, and low water holding capacity are major factors in achieving a satisfactory yield.

The impacts of N, water, and grain yield for maize were previously explained by Hammad, et al. [17] using quadratic equations. Ata-Ul-Karim, et al. [18,19] used thin-plate splines to evaluate the interaction between N application rates, soil characteristics, and Cd uptake in wheat grains. However, no attempt has been made to examine the interactive effects of cultivars, N application rates, and weather conditions under rainfed conditions, particularly for the Pothowar Plateau. Thus, complex and unclear genotypic, environmental, and management interactions (wheat cultivars, fluctuating weather conditions, and N rates) with contrasting findings necessitate a comprehensive study to understand better the fundamental agronomical and eco-physiological mechanisms affecting grain yield in wheat. Recently, Cossani and Sadras [16] reported that three-way interactions between water, N, and high temperature are poorly understood. We hypothesized that a favorable growing season would modify agronomic traits, WUE, and N use efficiencies, thereby promoting adequate uptake of available soil N.

Therefore, this study was conducted to investigate the effects of genotype, environment, and management practices and their interaction on agronomic traits, WUE, and N-use efficiencies under rainfed conditions and to explore the interaction of grain yield with agronomic traits, WUE, and N-use efficiencies under favorable and unfavorable growing conditions, to emphasize the critical role of N for crop growth.

## 2. Results

### 2.1. Weather Conditions

**Figure 1:**
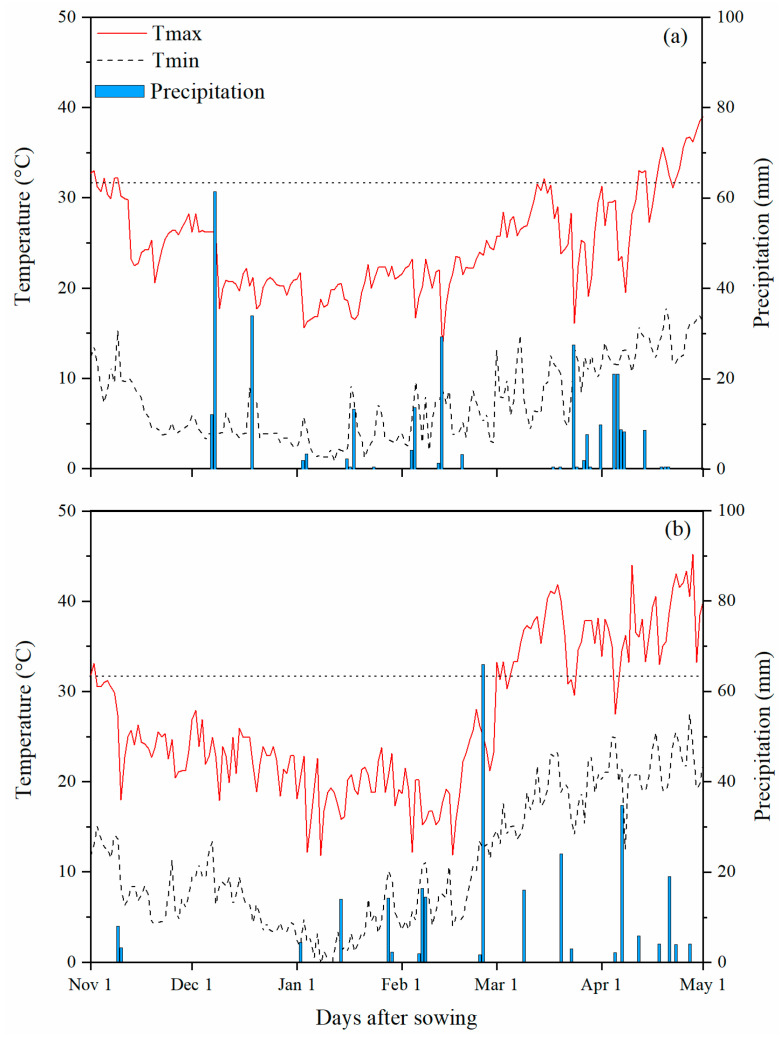
Weather data measured during wheat crop growing season for two years ((**a**); 2013–2014 and (**b**); 2014–2015) at Koont research farm. The dotted line represents the temperature threshold (>32 °C).

Precipitation was 360.8 mm and 292 mm during 2013–2014 and 2014–2015, respectively (Figure 1). The average minimum and maximum temperatures were 8.99 °C and 26.7 °C during 2013–2014, while they were 12.3 °C and 28.3 °C during the 2014–2015 growing season. Owing to the adequate and even rainfall (8% higher than mean rainfall in growing season) as well as ideal temperatures (Tmax < 32 °C) during 2013–2014, this season was considered as a favorable season for wheat growth and productivity. However, the wheat-growing season of 2014–2015 due to less rainfall (13% less rainfall than mean rainfall during growing season), particularly during the early vegetative growth period and elevated temperature (Tmax > 32 °C) during the reproductive phase was considered as unfavorable weather conditions for wheat growth and productivity.

### 2.2. Effect of Genotype, Weather, and Nitrogen Application Rates on Agronomic Traits

**Figure 2:**
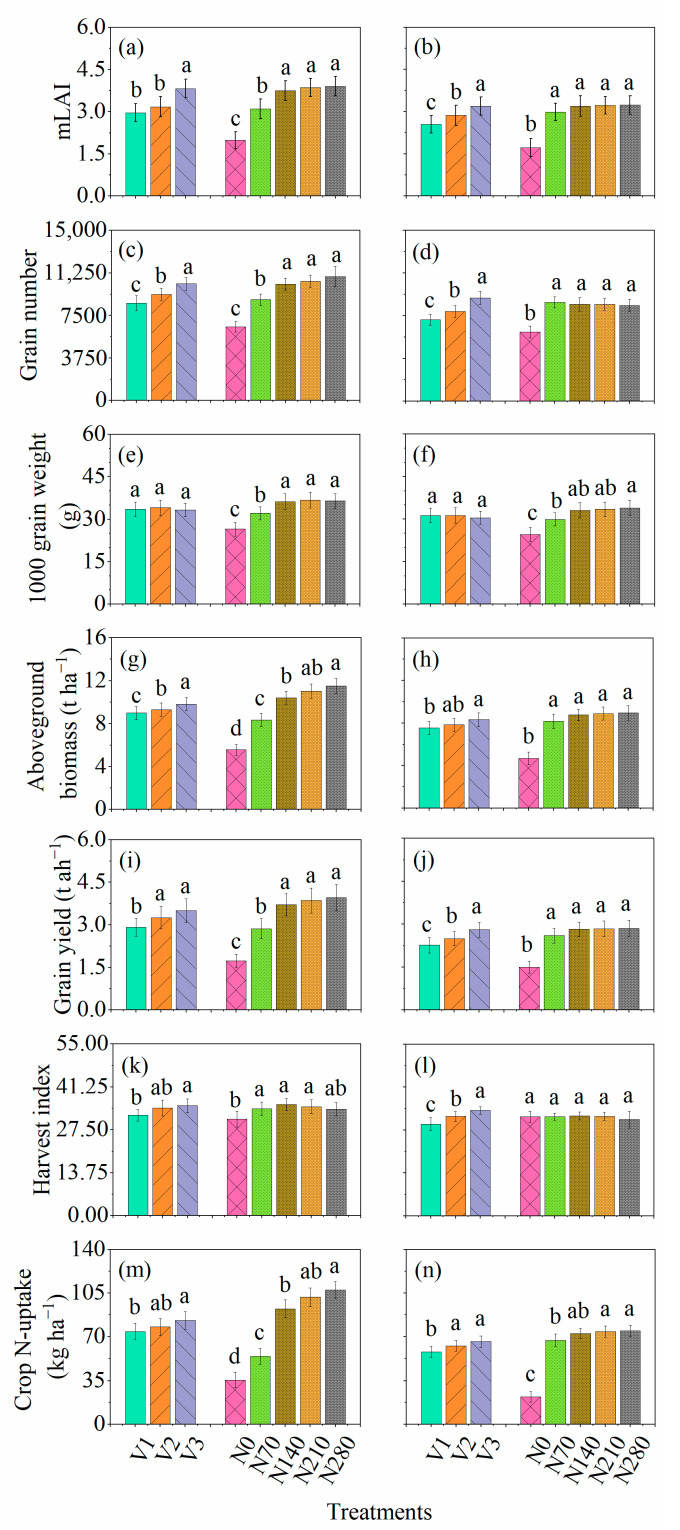
Response of (**a**,**b**) maximum leaf area index (mLAI), (**c**,**d**) grain number (m^−2^), (**e**,**f**) 1000 grain weight (g), (**g**,**h**) grain yield (t ha^−1^), (**i**,**j**) aboveground biomass (t ha^−1^), (**k**,**l**) harvest index, and (**m**,**n**) crop N-uptake (kg ha^−1^) for various cultivars and N application rates. The alphabets a, c, e, g, i, k, and m represent the first growing season (2013–2014), and b, d, f, h, j, l, and n represent the second growing season (2014–2015). V1, V2, and V3 represent AUR-809, CHK-50, and FSD-2008.

The ANOVA for the main effects and their interactions for maximum leaf area index (mLAI) and crop N-uptake are shown in Table 1. ANOVA indicated that mLAI and crop N-uptake was significantly affected by growing season, genotype, and N application rates. The mLAI showed no interactions between season × genotype, season × N application rates, genotype × N application rates, and season × genotype × N application rates. However, crop N-uptake showed significant interaction for season × N application rates. The mLAI and crop N-uptake ranged from 1.98 to 3.89 and 35.6 to 107.7 kg ha^−1^ across the cultivars and N application rates in the favorable growing season (2013–2014) while under the unfavorable growing season (2014–2015) it ranged from 1.72 to 3.27 and 22 to 74 kg ha^−1^. Minimum and maximum mLAI were observed for the AUR-809 and FSD-2008 during favorable and unfavorable growing seasons. Similar to mLAI, AUR-809 and FSD-2008 showed minimum and maximum crop N-uptake during the favorable and unfavorable growing seasons, respectively. Cultivars showed significant differences in both years for mLAI and crop N-uptake (Figure 2a,b,m,n). mLAI and crop N-uptake increased significantly from N_0_ to N_140_ and N_0_ to N_70_ during favorable and unfavorable growing seasons, respectively, while N additions beyond the aforementioned N rates posed no significant impacts on leaf area expansion (Figure 2a,b,m,n). Trend analysis of various cultivars for N application rates revealed that all cultivars showed linear and quadratic trends for mLAI and crop N-uptake (Table S1).

Grain number was influenced by five sources of variation: season, genotype, N application rates, season × N application rates, and genotype × N application rates. However, grain weight was significantly affected by season and N application rates, with no interaction effects. Grain number and grain weight ranged from 6512 to 10,661 and 26.6 to 36.8 g during the favorable growing season (Figure 2c,e) and ranged from 6073 to 9080 and 24.6.7 to 34.0 g during the unfavorable growing season (Figure 2d,f) depending upon various cultivars and N application rates. During the favorable and unfavorable growing season minimum and maximum grain number was recorded for AUR-809 and FSD-2008, respectively. In the case of grain weight, the minimum grain weight was recorded for AUR-809 and FDS-2008, and maximum grain weight was measured for CHK-50 during the favorable growing season. However, during the unfavorable growing season, minimum grain weight was measured for FSD-2008, and maximum grain weight was observed for CHK-50. A significant difference between cultivars was observed for both growing seasons for grain number, while grain weight showed no substantial difference (Figure 2c–f). For N application rates, grain number and grain weight improved significantly from N_0_ to N_140_ during the favorable growing season and N_0_ to N_70_ during the unfavorable growing season. All cultivars showed linear and quadratic increase in grain number and grain weight under N application rates.

The aboveground biomass, grain yield, and harvest index showed variation for four sources of variation (Table 1). ANOVA indicated that aboveground biomass, grain yield, and harvest index were significantly influenced by season, genotype, N application rates, and season × N application rates. The aboveground biomass, grain yield, and harvest index ranged from 5.57 to 11.5 t ha^−1^, 1.73 to 3.93 t ha^−1^, and 31 to 35.6 during the favorable growing season and ranged from 4.68 to 8.89 t ha^−1^, 1.49 to 2.84 t ha^−1^, and 29.4 to 33.7 unfavorable growing season, respectively. During the favorable growing season, minimum aboveground biomass, grain yield, and harvest index were recorded for AUR-809, while maximum aboveground biomass, grain yield, and harvest index were recorded for FSD-2008. The unfavorable growing season showed minimum aboveground biomass, grain yield, and harvest index for AUR-809. Maximum aboveground biomass, grain yield, and harvest index were shown for FSD-2008 under unfavorable growing season. There was a significant difference between cultivars for both growing seasons (Figure 2g–l). For N application rates, aboveground biomass and grain yield increased significantly up to N_140_ and N_70_, respectively, during favorable and unfavorable growing seasons, and no further difference between N_140_–N_280_ and N_70_–N_280_ was observed during favorable and unfavorable growing seasons (Figure 2g–l). In the case of harvest index, N application rates differed significantly from N_0_–N_70_ during the favorable growing season, while there was no significant difference during the unfavorable growing season (Figure 2k,l). Trend analysis showed variations in response of cultivars under N application rates for aboveground biomass, grain yield, and harvest index. AUR-809 and FSD-2008 represent linear and quadratic increase in aboveground biomass, grain yield, and harvest index under various N application rates. However, CHK-50 showed significant linear and quadratic increase for aboveground biomass and grain yield and showed no trend for harvest index (Table S1).

### 2.3. Effect of Genotype, Weather, and Nitrogen Application Rates on Water and Nitrogen Use Efficiencies

**Figure 3:**
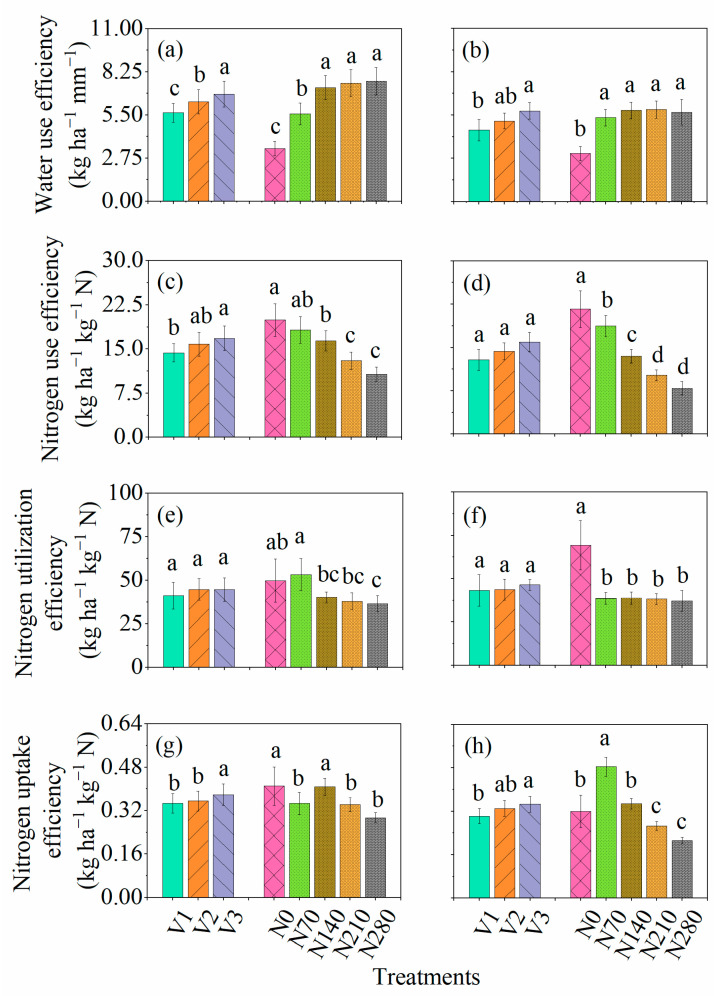
Response of (**a**,**b**) water use efficiency (kg ha^−1^ mm^−1^), (**c**,**d**) nitrogen use efficiency (kg ha^−1^ kg^−1^ N), (**e**,**f**) nitrogen utilization efficiency (kg ha^−1^ kg^−1^ N), and (**g**,**h**) nitrogen uptake efficiency (kg ha^−1^ kg^−1^ N) various cultivars and N application rates. The alphabets a, c, e, and g represent the first growing season (2013–2014), and b, d, f, and h represent the second growing season (2014–2015). V1, V2, and V3 represent AUR-809, CHK-50, and FSD-2008.

Water use efficiency (WUE) was influenced by four sources of variation: season, genotype, N application rates, and season × N application rates. However, season × genotype, genotype × N application rates, and season × genotype × N application rates have no significant impact on WUE. WUE ranged from 3.37 to 7.66 kg ha^−1^ mm^−1^ during the favorable growing season, while WUE ranged from 3.06 to 5.85 kg ha^−1^ mm^−1^ during the unfavorable growing season. Minimum and maximum WUE were shown by AUR-809 and FSD-2008, respectively, for both favorable and unfavorable growing seasons. Cultivars showed significant differences in both years (Figure 3a,b). N application substantially improved WUE from N_0_–N_140_ and N_0_–N_70_ during the favorable and unfavorable growing season (Figure 3a,b). In the case of trend analysis, all cultivars show significant linear and quadratic trend of increase in WUE with increasing N application rates.

For N use efficiency (NUE) and N uptake efficiency (NUpE), ANOVA indicated that NUE and NUpE were substantially affected by season, genotype, N application rates, and season × N application rates. Yet, N utilization efficiency (NUtE) showed variation for N application rates and season × N application rates. The NUE, NUtE, and NUpE ranged from 10.7 to 20 kg ha^−1^ kg^−1^ N, 36.5 to 53.3 kg ha^−1^ kg^−1^ N, and 0.29 to 0.41 kg ha^−1^ kg^−1^ N during favorable growing season and ranged from 7.91 to 21.7 kg ha^−1^ kg^−1^ N, 37.2 to 69.3 kg ha^−1^ kg^−1^ N, and 0.21 to 0.48 kg ha^−1^ kg^−1^ N during unfavorable growing season, respectively. The minimum NUE, NUtE, and NUpE were observed for AUR-809 for both growing seasons. While maximum NUE, NUtE, and NUpE were shown by FSD-2008 during both favorable and unfavorable growing seasons. During the favorable growing season, cultivars showed significant differences for NUE and NUpE, while NUtE showed non-significant differences between cultivars during both growing seasons (Figure 3c–h). For N application rate, maximum NUE was shown by N_0_ for both growing seasons, which significantly differs from N_140_–N_280_ during the favorable growing season, and from N_70_–N_280_ during the unfavorable growing season. NUtE showed a statistically higher value at N_70_ during the favorable growing season and at N_0_ during the unfavorable growing season. For NUpE, there was no significant difference between N_0_ and N_140_ during the favorable growing season. However, during the unfavorable growing season, N_70_ showed statistically higher NUpE and differed from other N application rates. Table S1 showed the significant linear trend of all cultivars for NUE under N application rates. For NUpE and NUtE, all cultivars showed linear and quadratic trend for various N application rates.

### 2.4. Interaction of Agronomic Traits, Water Use Efficiency, and Nitrogen Efficiencies Using Thin Plate Smoothing Spline

**Figure 4:**
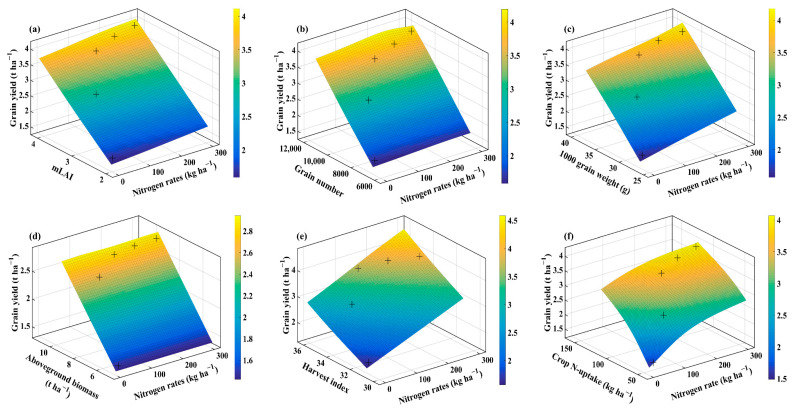
Thin plate smoothing spline plots showing the interaction of N rates (*x*-axis) with mLAI, grain number, 1000 grain weight, aboveground biomass, harvest index, and crop N-uptake (*y*-axis) on average grain yield (*z*-axis) in 2013–2014 growing season. The various panels show mLAI (**a**); grain number (**b**); 1000 grain weight (**c**); aboveground biomass (**d**); harvest index (**e**); and crop N-uptake (**f**) for the 2013–2014 growing season, respectively. + indicates the observed data.

**Figure 5:**
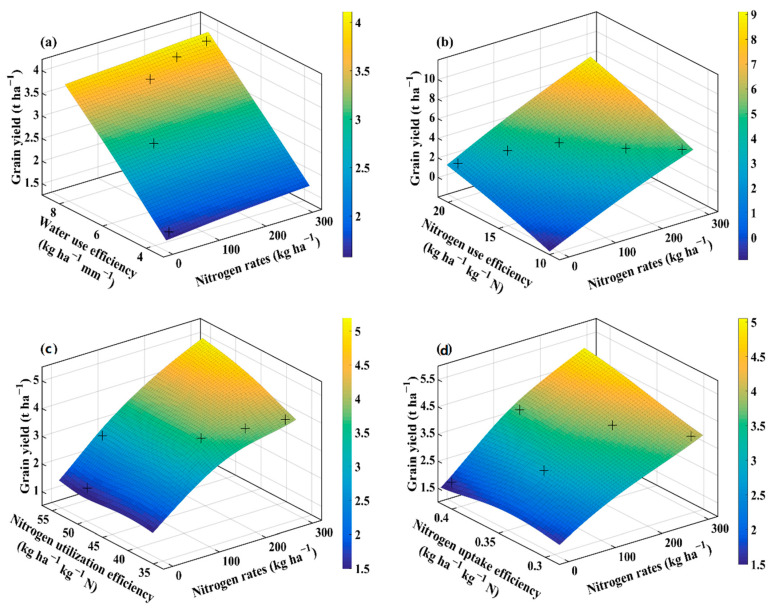
Thin plate smoothing spline plots showing the interaction of N rates (*x*-axis) with water use efficiency, nitrogen use efficiency, nitrogen utilization efficiency, and nitrogen uptake efficiency (*y*-axis) on average grain yield (*z*-axis) in the 2013–2014 growing season. The various panels show water use efficiency (**a**); nitrogen use efficiency (**b**); nitrogen utilization efficiency (**c**); and nitrogen uptake efficiency (**d**) for the 2013–2014 growing season, respectively. + indicates the observed data.

**Figure 6:**
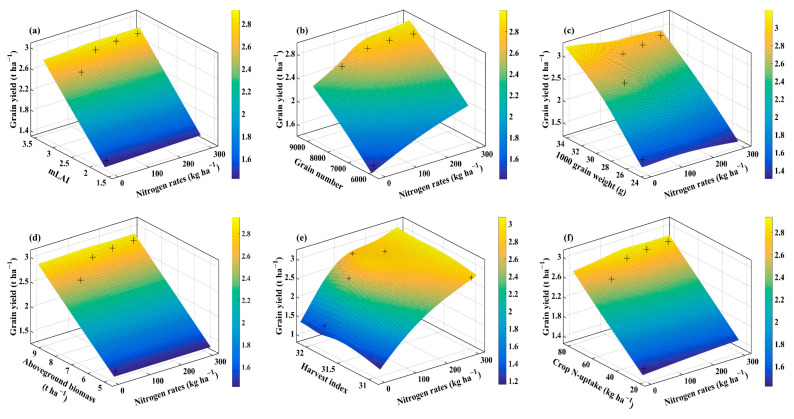
Thin plate smoothing spline plots showing the interaction of N rates (*x*-axis) with mLAI, grain number, 1000 grain weight, aboveground biomass, harvest index, and crop N-uptake (*y*-axis) on average grain yield (*z*-axis) in 2014–2015 growing season. The various panels show mLAI (**a**); grain number (**b**); 1000 grain weight (**c**); aboveground biomass (**d**); harvest index (**e**); and crop N-uptake (**f**) for the 2014–2015 growing season, respectively. + indicates the observed data.

**Figure 7:**
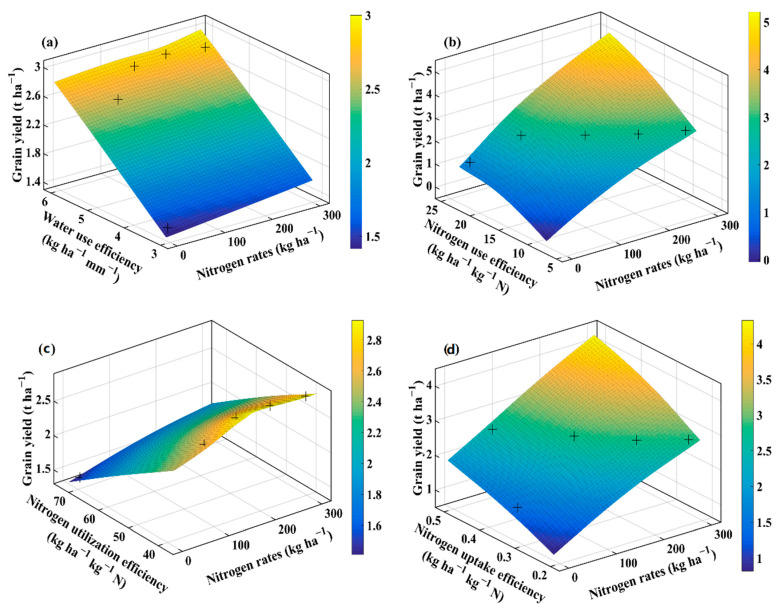
Thin plate smoothing spline plots showing the interaction of N rates (*x*-axis) with water use efficiency, nitrogen use efficiency, nitrogen utilization efficiency, and nitrogen uptake efficiency (*y*-axis) on average grain yield (*z*-axis) in the 2014–2015 growing season. The various panels show water use efficiency (**a**); nitrogen use efficiency (**b**); nitrogen utilization efficiency (**c**); and nitrogen uptake efficiency (**d**) for the 2014–2015 growing season, respectively. + indicates the observed data.

The thin plate smoothing spline plots were generated to investigate the interactive effects of agronomic traits, WUE, NUE, NUtE, NUpE, and N application rates on averaged wheat grain yield for the three cultivars (Figure 4, Figure 5, Figure 6 and Figure 7). The interactions of N application rates (*x*-axis), agronomic traits, WUE, NUE, NUtE, NUpE (*y*-axis), and grain yield (*z*-axis) are shown in Figure 4, Figure 5, Figure 6 and Figure 7. The thin plate smoothing spline plots represent the interactive and simultaneous effects of various N application rates, agronomic traits, water, and N use efficiencies on wheat grain yield. The impacts of N application and agronomic traits, WUE, NUE, NUtE, and NUpE on grain yield were evident in both growing seasons, and their interactive effects on grain yield were dependent on each other. Increasing N application rates, mLAI, grain number, plant aboveground biomass, harvest index, crop N-uptake, and WUE, resulted in higher grain yield for the two growing seasons. Generally, the blue color at the bottom of the plots depicted the lower grain yield, while the yellow color on the upper side represents the higher grain yield. The minimum grain yield was observed in the lower right-left corner (mLAI-N, grain number-N, grain weight-N, aboveground biomass-N, and WUE-N), lower-left corner (harvest index-N, crop N-uptake-N, NUE, NUtE, and NUpE) of the plots during 2013–2014. However, the maximum grain yield was observed in the upper right corner (grain number-N, grain weight-N, aboveground biomass-N, crop N-uptake-N, WUE, and NUtE) and upper left corner (mLAI-N, harvest index-N, NUE, and NUpE) of the plots during 2013–2014 growing season. Overall, increment in grain yield was more evident during the 2013–2014 growing season, and it ranged from 1 to 4.5 t ha^−1^ compared to those (1.4 to 3 t ha^−1^) in the 2014–2015 growing season. The minimum grain yield was observed in the lower left to right (mLAI-N, grain weight-N, plant aboveground biomass, and crop N-uptake) and left corner (grain number-N, harvest index-N, WUE-N, NUE-N, NUtE-N, and NUpE-N) of the plots during 2014–2015 (Figure 6 and Figure 7). The highest values of grain yield were observed in the upper left corners (grain weight-N, plant aboveground biomass-N, N-uptake-N, WUE-N, and NUpE-N) and upper right corner (mLAI-N, grain number-N, harvest index-N, NUE-N, and NUtE-N) of the plots.

## 3. Discussion

Water, N, and elevated temperature during crop ontogeny are the major constraints for the rainfed cropping system [16]. Erratic and scarce rainfall during the entire crop growth period makes the matching of N supply to crop demand and available water supply more challenging under rainfed areas [8,20]. Consequently, it is imperative to quantify the simultaneous impacts of water, N, and high temperatures on wheat growth and productivity [21]. Therefore, this study investigated the effects of N application rates on the development and productivity of three wheat cultivars in seasons with contrasting climatic conditions (Favorable: with optimal rainfall and temperature) and (Unfavorable: with uneven and low rainfall and high temperature).

Agronomic traits have been reported to be affected by water, N, and heat stress [16]. The alleviation of the adverse effects of water scarcity and high temperature through the optimized use of N in this study was in agreement with previous reports [22,23]. Low rainfall and higher temperatures during unfavorable growing seasons negatively impacted mLAI, aboveground biomass, grain number, grain size, and grain yield [24]. The alleviation of adverse effects of drought and high temperature in this study was accredited to the enhanced stomatal conductance, chlorophyll content, net photosynthetic rate, transpiration rate, intercellular CO_2_, mLAI, grain number, grain size, and root growth as a consequence of N fertilization application [22,23]. A long-term study conducted to evaluate the impact of agroclimatic extremes on the performance of wheat cultivars revealed that only a few cultivars tolerated the adverse agroclimatic conditions [25]. In the present study, FSD-2008 performed better across various N application rates and weather conditions. Besides, the better response of FSD-2008 to N application than other cultivars might potentially be associated with its large sink size (grain size and grain number), indicating the feedback mechanism between N-uptake and sink size [26]. The overall significant increase in agronomic traits, WUE, NUE, NUtE, and NUpE from N_0_–N_140_ and N_0_–N_70_ during favorable and unfavorable growing conditions could be elucidated by the fact that wheat grain yields were responsive to higher N application rates when rainfall was optimum and evenly distributed. Asseng, et al. [13] similarly observed that in moderate and high rainfall zones, the magnitude of response depended on seasonal rainfall distribution.

The water use efficiency of wheat has been reported to be highly variable under field conditions [13,27], with the average WUE ranging from 9.9 to 5.3 kg grain ha^−1^mm^−1^ across environments [28]. Crop management practices that reduce the soil water evaporation and increase N-uptake by crop plants can be used to enhance WUE at the canopy level. Due to the differences in soil water-holding capacity, soil evaporation, their interactions, rainfall patterns, and management practices, the soil type plays a decisive role in the crop WUE and NUE [13]. In a favorable growing season, higher WUE results in higher grain yield than in an unfavorable growing season. The seasonal variability in grain yield and yield components can be attributed to the simultaneous effects of the magnitude and distribution of rainfall throughout the growing season. For example, Sadras, et al. [20] reported that wheat grain yield responded to summer rainfall as a function of growing conditions, specifically rainfall during the growing season. In the present study, the summer rainfall was high during the favorable growing season leading to high initial soil moisture than under the unfavorable growing season. Patanita, et al. [29] also found higher grain yield during favorable growing season (adequate and timely rainfall) and lower grain yield during unfavorable growing season (extreme aridity). Further, the close association between WUE and N application rates in determining the grain yield under rainfed conditions in this study was in concession with previous reports [30,31]. Apart from its direct effect on grain yield and aboveground biomass production, water supply also interacts with N supply to enhance NUE by optimizing crop N uptake [32]. A significant positive correlation of grain yield was found between agronomic traits and WUE. However, a significant negative correlation of grain yield was observed with NUE and NUtE, whereas NUpE had a non-significant negative relationship with grain yield (Table S2). These findings are supported by previous studies by Yousaf, et al. [33], Robertson and Kirkegaard [34] and Xu, et al. [35], who find positive correlation between grain yield and agronomic traits, and negative correlation between N use efficiencies. N use efficiencies showed variability for weather conditions, cultivars, and N application rates. Overall, NUE, NUtE, and NUpE were high during the favorable growing season due to even and adequate rainfall throughout the growing season. Qadeer, et al. [22] also found a significant variation in NUE, NUtE, and NUpE under different locations and N application rates. Todeschini, et al. [36] noted a genotypic variation for N use efficiencies. Higher NUE, NUtE, and NUpE were recorded for FSD-2008 which attributed the variability of cultivar response to NUE, which is a function of NUtE, NUpE, N remobilization efficiency [36]. Additionally, leaf chlorophyll content and aboveground biomass accumulation are traits linked to NUE and can thus be used for indirect selection of N responsive cultivars [36]. The results of NUtE and NUpE are in accordance with Qadeer, et al. [22] showing a decreasing trend with increased N application rates, whereas NUpE increased up to certain N application rates and then decreased. The disparity in the behavior of cultivars under different N levels in relation to the NUtE is important for cultivar selection and management practices [36].

The findings confirmed the simultaneous role of N application rates, agronomic traits, WUE, NUE, NUtE, and NUpE for optimizing grain yield under particular agroclimatic conditions. The interactions between agronomic traits, WUE, NUE, NUtE, and NUpE, were more evident during favorable conditions than unfavorable conditions, due to even and adequate rainfall, suitable initial soil moisture along with optimum temperature prevailed that during the favorable growing season compared to the unfavorable growing season having low rainfall during the vegetative phase and high temperatures during the reproductive phase. The compensation of grain yield (72% increase) with N application (N_0_ to N_70_) under unfavorable agroclimatic conditions in this study corroborates with a previous study showing that N application rates under deficit irrigation conditions (50% field capacity) compensate 62.3% of grain yield [17]. Among interactions, growing season × N application rates showed significant variation with respect to the majority of agronomic traits and resource use efficiencies which means that favorable growing conditions are critical to capture the benefits of applied N. Further, in the case of the thin-plate spline plots, we averaged the cultivar data as cultivars did not show significant interaction for growing season × cultivar. The thin plate spline plots showed higher grain yield during the favorable growing season than unfavorable growing season as N mineralization dynamics, N losses, crop growth, and N uptake strongly depend on the amount and distribution of rainfall and temperatures that prevailed during the growing season [37].

Sustainable grain production is the primary objective of current agronomic research, which could be accomplished by using appropriate management practices that provide sufficient N to meet crop demand while conserving soil and water quality [38]. Thus, optimizing NUE is critical from an environmental, agricultural, and economic perspective. Despite the fact that crop NUE has steadily improved over the years in tandem with crop yield [39], more than half of applied N is not utilized by the crops worldwide [40]. Unused N in soil is a concern for the environment due to atmospheric release and leaching. Further, there is a significant difference in NUE among crops, regions, and cropping systems. Thus, both crop-specific and local solutions should be considered to improve NUE at a local and global scale [41]. Hence, agronomists and breeders must conduct comprehensive studies in contrasting environments using different genotypes to identify opportunities for improving NUE in crops. For example, during the wetter season, optimum N application rates increased compared to the dry season. Schmidt, et al. [42] discovered that the optimal N application rate for maize increases as available soil water increases and that year-to-year variation in maize yield was mainly due to moisture variability in July. Thus, weather forecasting may assist in determining the optimal amount of N to apply, resulting in increased NUE.

A recent study conducted in the loess plateau of China reported that wheat yield increased with N application under favorable agroclimatic conditions. However, the N application should be optimized according to prevailing conditions to avoid any N loss under unfavorable agroclimatic conditions [43]. In the current study, the increase in grain yield was only 10% from N_70_ to N_280_ under unfavorable growing season. In rainfed areas, N should be applied a little earlier than the expected rainfall. Meteorological forecasting can assist in estimating the time of N application. Consequently, N management strategies should be tailored considering cultivars, target yields, and agroclimatic conditions in rainfed cropping systems. Our results demonstrated the G×E×M interactions that help policymakers and farmers choose appropriate wheat cultivars and N application rates by utilizing meteorological forecasting, summer rainfall, and initial soil moisture content.

In our study, N application rates did not respond to agronomic traits, WUE, and N uses efficiencies under unfavorable agroclimatic conditions. Adequate and even rainfall (water supply) and optimal temperature are required to take advantage of additional N; this demonstrates the resource co-limitation for wheat production in these environments. Since increased temperatures and droughts may increase N losses, new opportunities for enhancing crop yields must be explored. Thus, future studies should consider various cultivars, locations, and N application timings based on rainfall and crop modeling for examining the genotypic potential of wheat cultivars and N application rates under diverse agroclimatic conditions and future climate scenarios in rainfed areas.

## 4. Materials and Methods

### 4.1. Field Experiments

Field experiments were conducted at the Koont Research Farm, Chakwal (32°93′ N, 72°86′ E), a research site of PMAS-Arid Agriculture University Rawalpindi during wheat growing seasons of 2013–2014 and 2014–2015. Geographically Chakwal is located in the Pothowar Plateau of Punjab, Pakistan, and is 498 m above mean sea level. The climate of Chakwal is characterized as semi-arid. The summer season begins in April and lasts until October. May and June are the hottest months, with daytime temperatures typically ranging from 40 to 45 °C. The winter season begins in November and lasts until March. January is the coldest month, with a mean minimum temperature of 1 °C. The average total precipitation, minimum, and maximum temperatures during 2013–2015 were 637.4 mm, 15.0 °C, and 30.2 °C.

Two-year experiments were conducted with three recommended high input responsive spring wheat cultivars (FSD-2008 = Faisalabad-2008, CHK-50 = Chakwal-50, and AUR-809) for semi-arid regions of Pothowar Plateau and five N levels (N_0_ = controlled, N_70_ = 70 kg ha^−1^, N_140_ = 140 kg ha^−1^, N_210_ = 210 kg ha^−1^, N_280_ = 280 kg ha^−1^) with three replicates. The cultivar FSD-2008 was developed by Ayub Agricultural Research Institute, Faisalabad. CHK-50 was developed by Barani Agricultural Research Institute, Chakwal. The cultivar AUR-809 was developed by Department of Plant Breeding, PMAS-Arid Agriculture University. The potential grain yield of FSD-2008, CHK-50, and AUR-809 was recorded as 6000, 4000, and 3300 kg ha^−1^, respectively, during national trials. Urea, P_2_O_5_ (100 kg ha^−1^), and K_2_O (30 kg ha^−1^) fertilizers were applied as basal fertilizers at the time of sowing in both seasons. The experiments were laid out in randomized complete block design (RCBD) with split plot arrangements having cultivars in the main plot and N levels in the subplot. The net plot size was set as 5 × 8 m^2^. The total number of treatments was 30 with three replications (3 cultivars × 5 N levels × 2 years = 30). The wheat cultivars were sown on 13th November for consecutive years with a row-to-row distance of 25 cm. Sowing was done with a sowing drill using recommended seed rate of 120 kg ha^−1^ for the rainfed area. Weeding was done manually to control weeds. All other management practices were maintained in accordance with local authority recommendations in all treatments.

### 4.2. Weather and Soil Data

Daily meteorological data for Koont Research Farm, including solar radiation, maximum and minimum temperatures, and rainfall, were obtained from the Pakistan Meteorological Department (Figure 1). The soil was sampled at 0–15, 15–30, 30–45 cm using a King tube, and soil parameters are presented in Table 2. The 15-year average of seasonal (from November to May) minimum and maximum temperature and rainfall were 10 ± 3 °C, 23 ± 5 °C, and 336 ± 40 mm, respectively. The 2013–2014 growing season was declared favorable due to adequate and timely rainfall and optimum temperature during the entire growing season. Conversely, the 2014–2015 growing season was stated as unfavorable growing season due to lack of precipitation during early vegetative phase and heat stress during reproductive phase (Figure 1).

Soil pH and electrical conductivity (EC) were estimated at soil to deionized water (without CO_2_) ratio of 1:2.5 (*w*/*v*) and 1:5 (*w*/*v*) using Thermo Scientific Orion 4-star meter and EC meter, respectively. Soil moisture content was measured by the gravimetric method by determining dry soil mass in each core [44]. N, available phosphorous (P), and potassium (K) were measured by using the methods described by Bremner and Mulvaney [45], Olson, et al. [46], and Richards [47]. Soil organic carbon was calculated by Walkley and Black [48] approach.

### 4.3. Measurement of Crop Parameters

Maximum leaf area index (mLAI, ratio of leaf area to land area), grain number (m^−2^), 1000 grain weight (g), aboveground biomass (t ha^−1^), grain yield (t ha^−1^), harvest index (ration between grain yield and aboveground biomass), crop N-uptake (kg ha^−1^), water use efficiency (WUE), N use efficiency (NUE, kg ha^−1^ kg^−1^ N), N-utilization efficiency (NUtE, kg ha^−1^ kg^−1^ N), N-uptake efficiency (NUpE, kg ha^−1^ kg^−1^ N) were recorded during both growing seasons. mLAI was measured manually at heading stage by the method described by Amanullah, et al. [49] and Ahmad, et al. [50]. Aboveground biomass refers to top ground biomass excluding roots. Phenology of wheat plants was monitored by using Zadoks scale [51]. All wheat cultivars used in this study were medium duration maturity cultivars. All cultivars were harvested at the end and mid of April for favorable and unfavorable growing seasons, respectively. Wheat plants were harvested from an area of 1m^2^ from each plot at two randomly selected locations at physiological maturity to calculate the grain number and grain yield. A total of 1000 grain weight (g) was taken at random from threshed samples of grain yield, and the grains were manually counted and weighed. For the crop N-uptake, harvested wheat plants were oven-dried for 48 h at 68 °C. The Micro-Kjeldahl method was used to calculate total N concentration of plants [52]. The crop N-uptake was determined as the sum of N accumulation in plants. WUE was calculated as follows [43],

(1) WUE=Grain yield/ET

where ET is evapotranspiration and water balance method was used to estimate the ET as follows [43],

(2) ET=P+ΔSWS−R−D

where P denotes the effective rainfall, more than 5 mm; ∆SWS denotes the change in soil water storage during a period, mm; R denotes surface runoff (mm), which was omitted in this study due to the flat surfaces and high ridges of the experimental plots. D is the deep drainage (mm), which was similarly overlooked because practically all of the rainfall that penetrated the soil in this location was held at the 0–50 cm soil depth. As a result, ET was determined in the following manner:

(3) ET=P+ΔSWS

Qadeer, et al. [22] procedure was applied to calculate the NUE, NUtE, and NUpE:

(4) $$\mathrm{NUE}=\frac{\mathrm{Grain}\;\mathrm{yield}}{N_{\mathrm{supply}}}$$

(5) $$\mathrm{NUtE}=\frac{\mathrm{Grain}\;\mathrm{yield}}{\mathrm{N-uptake}}$$

(6) $$\mathrm{NUpE}=\frac{\mathrm{N-uptake}}{N_{\mathrm{supply}}}$$

where N_supply_ is the sum of soil N content at sowing and applied N fertilizer, N-uptake is crop N-uptake.

### 4.4. Statistical Analysis

The analysis of variance (ANOVA) was performed on the data from two growing seasons (2013–2014 and 2014–2015) for agronomic traits, WUE, and N uses efficiencies of three spring wheat cultivars at five different N rates using split-plot AOV on Statistics 10.0 software (USA). Tukey’s HSD was used for posthoc comparisons at the 95% level of significance. Orthogonal contrasts were performed on pooled data to determine the significant linear, quadratic, and cubic response of each cultivar under varying N application rates. Pearson correlation was used to analyze direct relationships between grain yield and agronomic traits, WUE, and N use efficiencies for favorable and unfavorable growing season. The interactions (response surface plots) between grain yield, N application rates, agronomic traits, WUE, and N use efficiencies were established using a thin-plate smoothing spline in MATLAB (The MathWorks, Inc., Natick, Massachusetts). Thin plate splines are smoothing splines used to illustrate complex interactions between response variables and continuous predictors. Thin plate splines are well-suited for examining the combined effects of two continuous predictors on a single result due to their multi-dimensional appearance. Instead of having a single curve, thin-plate splines have a bendable surface. Each continuous variable is represented on its *x*-axis, yielding a bivariate surface in two dimensions.

## 5. Conclusions

Our findings emphasize the significance of G × M × E interactions for optimizing the grain yield of wheat grown under rainfed conditions. Our results highlight the importance of N in wheat adaptation to low rainfall environments and the potential for further yield improvement through resource-integrated traits. The responses to various variables are range-dependent, so inferences must be context-specific; caution is advised when drawing broad conclusions, such as the wheat cultivars and N application compounds’ impact on unfavorable growing seasons. Wheat cultivars responded differently to applied N under rainfed conditions. Wheat growth and development, WUE, NUE, and NUpE were significantly influenced by season, genotype, N application rates, and season × N application rates. However, NUtE was substantially affected by N application rates and interaction of season × N application rates. The grain yield of FSD-2008 was the highest in both growing seasons among the cultivars used in this study. Among N application rates, N_140_ and N_70_ showed higher grain yields for the favorable and unfavorable growing seasons. Thus, co-limitation of water and N should be considered to maximize the grain yield under rainfed environments. The optimal N application rate is the key to achieving maximum grain yield by optimizing agronomic traits and resource (N and water) efficiencies under favorable and unfavorable growing conditions. To achieve high grain yields, additional locations, cultivars, and management practices should be investigated in the future study.

## Figures and Tables

**Table 1:** Agronomic traits, water use efficiency, and N use efficiencies of the wheat crop during 2013–2015.

	mLAI	Grain Number	1000 Grain Weight	Grain Yield	Aboveground Biomass	Harvest Index	Crop N-Uptake	WUE	NUE	NUtE	NUpE
			g	t ha^−1^	t ha^−1^		kg ha^−1^	kg ha^−1^ mm^−1^	kg ha^−1^ kg^−1^ N	kg ha^−1^ kg^−1^ N	kg ha^−1^ kg^−1^ N
Season (S)	***	***	***	***	***	***	***	***	**	ns	***
Genotype (G)	***	***	ns	***	***	***	***	***	***	ns	***
Nitrogen (N)	***	***	***	***	***	*	***	***	***	***	***
S × G	ns	ns	ns	ns	ns	ns	ns	ns	ns	ns	ns
S × N	ns	***	ns	***	***	*	***	***	**	***	***
G × N	ns	**	ns	ns	ns	ns	ns	ns	ns	ns	ns
S × G × N	ns	ns	ns	ns	ns	ns	ns	ns	ns	ns	ns

mLAI: maximum leaf area index, WUE: water use efficiency, NUE: nitrogen use efficiency, NUtE: nitrogen utilization efficiency, NUpE: nitrogen uptake efficiency. * *p* < 0.05, ** *p* < 0.01, *** *p* < 0.001.

**Table 2:** Averaged soil physiochemical analysis for two years (2013–2014 and 2014–2015) experiments conducted at Koont research farm.

Soil Properties	Depth (cm)		
	0–15	15–30	30–45
pH	8.15	8.35	8.4
EC (dS m^−1^)	0.3	0.325	0.285
Nitrogen (%)	0.035	0.19	0.175
Nitrate-N (mg Kg^−1^)	3.55	3.49	3.34
AV. P (mg Kg^−1^)	2.59	2.775	2.645
K (mg Kg^−1^)	111.5	146.5	153
Organic C (%)	0.7	0.46	0.44
Silt (%)	23	21	20
Sand (%)	56	56	56
Clay (%)	21	23	24
Texture	Sandy clay loam	Sandy clay loam	Sandy clay loam
B. Density (g cm^−3^)	1.3	1.565	1.655
SLL (mm mm^−1^)	0.059	0.078	0.078
SDULL (mm mm^−1^)	0.27	0.22	0.215
Saturated SW (mm mm^−1^)	0.38	0.34	0.3

AV. P: available phosphorus, B. Density: bulk density, SLL: soil lower limit, SDUL: soil drain upper limit, and SW: soil water.

## Data Availability

All data generated or analyzed during this study are included in this article.

## Supplementary Materials

The following are available online at https://www.mdpi.com/article/10.3390/plants10112310/s1, Table S1: Trend analysis of pooled data of two growing seasons for the effect of N application rates on agronomic traits†, water use efficiency, and N traits‡ of three cultivars. Table S2: The correlation of wheat grain yield with agronomic traits, water use efficiency (WUE), nitrogen use efficiency (NUE), nitrogen utilization efficiency (NUtE) and nitrogen uptake efficiency (NUpE) under favorable and unfavorable growing conditions.
