# Supplementary material for: Exploring the Impacts of Genotype-Management-Environment Interactions on Wheat Productivity, Water Use Efficiency, and Nitrogen Use Efficiency under Rainfed Conditions
Source: Plants (Basel). 2021 Oct 27;10(11):2310. doi: 10.3390/plants10112310 (PMC8623311; doi:10.3390/plants10112310)
Supplement: Supplementary file 1 [file plants-10-02310-s001.zip › plants-1346352-supplementary.pdf]

Supplementary Table S1. Trend analysis of pooled data of two growing seasons for the effect of N application rates on agronomic traits†, water use efficiency, and N traits‡ of three cultivars.

Cultivars	Trend analysis	mLAI	Grain number	1000 grain weight g	Grain yield t ha^{-1}	Aboveground biomass t ha^{-1}	Harvest index	Crop N-uptake kg ha^{-1}	WUE⸸ kg ha^{-1} mm^{-1}	NUE kg ha^{-1} kg^{-1} N	NUtE kg ha^{-1} kg^{-1} N	NUpE kg ha^{-1} kg^{-1} N
AUR-809	Linear	***	***	***	***	***	ns	***	***	***	***	***
	Quadratic	***	**	**	**	**	*	**	**	ns	ns	*
	Cubic	ns	ns	ns	ns	ns	ns	ns	ns	ns	ns	ns
CHK-50	Linear	***	***	***	***	***	ns	***	***	***	***	***
	Quadratic	***	**	**	**	***	ns	**	**	ns	*	*
	Cubic	*	ns	ns	ns	ns	ns	ns	ns	ns	ns	ns
FSD-2008	Linear	***	***	***	***	***	*	***	***	***	***	***
	Quadratic	***	***	***	**	***	*	***	***	ns	*	**
	Cubic	ns	ns	ns	ns	ns	ns	ns	ns	ns	ns	ns

† mLAI, grain number, 1000 grain weight, grain yield, biomass, harvest index, and crop N-uptake.

⸸ WUE, water use efficiency

‡ NUE, nitrogen use efficiency, NUtE, nitrogen utilization efficiency, NUpE, nitrogen uptake efficiency.

* $p < 0.05$, ** $p < 0.01$, *** $p < 0.001$.

Supplementary Table S2. The correlation of wheat grain yield with agronomic traits, water use efficiency (WUE), nitrogen use efficiency (NUE), nitrogen utilization efficiency (NUtE) and nitrogen uptake efficiency (NUpE) under favorable and unfavorable growing conditions.

		mLAI	Grain number	1000 grain weight	Aboveground biomass	Harvest index	Crop N-uptake	WUE	NUE	NUtE	NUpE
Grain yield	Favorable growing season	0.97***	0.99***	0.93***	0.99***	0.8***	0.96***	1***	-0.69**	-0.69**	-0.42
	Unfavorable growing season	1***	0.93***	0.85***	0.98***	0.33	0.97***	0.99***	-0.63*	-0.84***	-0.05

* $p < 0.05$, ** $p < 0.01$, *** $p < 0.001$.
